# Metal release profiles of orthodontic bands, brackets, and wires: an in vitro study

**DOI:** 10.1007/s00056-017-0107-z

**Published:** 2017-09-14

**Authors:** B. Wendl, H. Wiltsche, E. Lankmayr, H. Winsauer, A. Walter, A. Muchitsch, N. Jakse, M. Wendl, T. Wendl

**Affiliations:** 1 Division of Oral Surgery and Orthodontics, Department of Dental Medicine and Oral Health, Medical University, Graz, Austria; 2 Institute of Analytical Chemistry and Radiochemistry, University of Technology, Graz, Austria; 3 Private Practice, Bregenz, Austria; 4 Departamento de Ortodoncia y Ortopedia Dento-facial, Universitat Internacional de Catalunya, Barcelona, Spain; 5 Institute of Biomedical Engineering, Technical University Graz, Graz, Austria

**Keywords:** Orthodontic materials, Metal release, Mass spectrometer, Corrosion, Biocompatibility, Allergy, Kieferorthopädische Materialien, Metallfreisetzung, Massenspektrometer, Korrosion, Biokompatibilität, Allergie

## Abstract

**Aim:**

The present study evaluated the temporal release of Co Cr, Mn, and Ni from the components of a typical orthodontic appliance during simulated orthodontic treatment.

**Materials and methods:**

Several commercially available types of bands, brackets, and wires were exposed to an artificial saliva solution for at least 44 days and the metals released were quantified in regular intervals using inductively coupled plasma quadrupole mass spectrometry (ICP-MS, Elan DRC+, Perkin Elmer, USA). Corrosion products encountered on some products were investigated by a scanning electron microscope equipped with an energy dispersive X-ray microanalyzer (EDX).

**Results:**

Bands released the largest quantities of Co, Cr, Mn, and Ni, followed by brackets and wires. Three different temporal metal release profiles were observed: (1) constant, though not necessarily linear release, (2) saturation (metal release stopped after a certain time), and (3) an intermediate release profile that showed signs of saturation without reaching saturation. These temporal metal liberation profiles were found to be strongly dependent on the individual test pieces. The corrosion products which developed on some of the bands after a 6-month immersion in artificial saliva and the different metal release profiles of the investigated bands were traced back to different attachments welded onto the bands.

**Conclusion:**

The use of constant release rates will clearly underestimate metal intake by the patient during the first couple of days and overestimate exposure during the remainder of the treatment which is usually several months long. While our data are consistent with heavy metal release by orthodontic materials at levels well below typical dietary intake, we nevertheless recommend the use of titanium brackets and replacement of the band with a tube in cases of severe Ni or Cr allergy.

## Zusammenfassung

**Zielsetzung:**

In dieser Studie wurde die Freisetzung von Co(Kobalt)-, Cr(Chrom)-, Mn(Mangan)- und Ni(Nickel)-Metallionen aus typischen kieferorthopädischen Apparaturen in einer künstlichen Speichellösung gemessen.

**Materialien und Methoden:**

Handelsübliche Bänder, Brackets und Drähte wurden für mindestens 44 Tage einer künstlichen Speichellösung ausgesetzt, und die freigesetzten Metalle wurden unter Verwendung eines induktiv gekoppelten Plasma-Quadrupol-Massenspektrometers (ICP-MS, Elan DRC+, PerkinElmer, USA) quantifiziert. Korrosionsprodukte wurden durch ein Rasterelektronenmikroskop mit einem Röntgenmikroanalysator (EDX) untersucht.

**Ergebnisse:**

Bänder setzten die größten Mengen an Co, Cr, Mn und Ni frei, gefolgt von Brackets und Drähten. Drei verschiedene Metallfreisetzungsprofile ließen sich beobachten: (1) eine konstante, wenn auch nicht lineare Freisetzung, (2) eine Sättigung (Metallfreisetzung stoppt nach einer bestimmten Zeit) und (3) ein intermediäres Freisetzungsprofil mit Zeichen einer Sättigung. Diese temporären Metallfreisetzungsprofile waren stark von den einzelnen Teststücken abhängig. Die Korrosionsprodukte, die sich nach einer 6-monatigen Exposition in künstlichem Speichel auf einigen der Bänder entwickelten, und deren verschiedene Metallfreisetzungsprofile wurden auf die aufgeschweißten Attachments der Bänder zurückgeführt.

**Schlussfolgerung:**

Bei Verwendung von konstanten Freisetzungsraten wird die Metallaufnahme durch den Patienten während der ersten paar Tage deutlich unterschätzt und die Exposition im Verlauf der sich anschließenden, i.d.R. etliche Monate dauernden Behandlung, überschätzt. Auch wenn die Konzentration der Schwermetallfreisetzung durch kieferorthopädische Materialien deutlich unterhalb der der typischen Nahrungsaufnahme liegt, empfehlen wir die Verwendung von Titan-Brackets und die Vermeidung von Bändern in Fällen von schwerer Ni oder Cr-Allergie.

## Introduction

The metallic components presently used in orthodontic treatment differ substantially with respect to their composition and characteristics, reflecting the different physical and mechanical properties required. The introduction of nickel–titanium alloys in 1971 for instance brought important progress for orthodontic treatment [2].Continuous long-term contact of orthodontic materials with teeth, gingivae, and the oral environment in general results in biodegradation of metals. Thereby, the patient is exposed to increased levels of metals and metal ions [13, 23] beyond those resulting from dietary intake and other forms of exposure. The average daily intakes of nickel and chromium in food are, for instance, estimated to be 200–300 µg nickel/day [8] and 30–100 µg chromium/day [21]. While titanium has been proven to be biocompatible, nickel is a known toxin and exposure must be carefully monitored [5, 22]. Nickel–titanium alloys generally have a nickel content of more than 50% compared to approximately 8% in steel wires [23]. Nickel is also a potential allergen [15] and can cause hypersensitivities, while nickel sulfide, oxide, and carbonate salts have also been classified as carcinogens [20]. Furthermore, orthodontic treatment may also increase the exposure to other heavy metals such as chromium, cobalt, and manganese. Manganese is a neurotoxin and can interfere with the central nervous system [27]. Special emphasis must also be given to the tracking of chromium, in particular Cr(VI) which is toxic and mutagenic as it can damage DNA [26]. Chromium can, moreover, also act as an allergen [6].

In general, many metallic materials are chemically resistant to corrosion as long as a surface oxide layer is present. As soon as the oxide layer dissolves, the onset of surface corrosion starts [4, 9, 13]. The corrosion rate is influenced by the composition of the material, the chemical and thermal environment, the surface area, and the degree of surface smoothness [16, 24].

The European Committee for Standardization devised via EN 1811:2011 a reference test method for the release of nickel from products intended to come into contact with skin, according to which nickel release must not exceed 0.5 µg/cm^2^/week [28].

The aim of the present study was to evaluate Co, Cr, Mn, and Ni release (amounts and temporal release patterns) by a typical orthodontic appliance during simulated orthodontic treatment by long-term exposure of a range of commercially available bands, brackets, and wires to an artificial saliva solution.

## Materials and methods

### Reagents

Purified water (18 MΩ cm, Barnstead Nanopur, USA) and high purity acids (HNO_3_ purified by subboiling in a quartz still) were used throughout. Co, Cr, Fe, Ni, and Mn calibration standards (0; 0.1; 0.4; 1; 10; 20 µg l^−1^) were prepared by diluting 1 g l^−1^ single element stock solutions (Alfa Aesar, Germany) in a mixture of 10% artificial saliva solution and 90% diluted nitric acid (0.6% HNO_3_ v/v). Y (Alfa Aesar, Germany) was used as internal standard.

The artificial saliva solution was prepared by dissolving 1.0 g urea (U5128-500G, Sigma Aldrich, Germany), 0.7 g NaH_2_PO_4_.2H_2_O (Reag Ph. Eur, Merck, Germany), 0.4 g NaCl (pa, Merck, Germany), and 0.4 g KCl (pa, Merck, Germany) in 1000 ml of high purity water.

### Preparation of the test pieces

A range of orthodontic bands, brackets, and wires from three different manufacturers were selected for investigation (see Table 1 for a list of the products used and the compositions provided, in each case, by the respective manufacturers). Grouped samples, consisting of either 4 bands, or 20 brackets or three wires from each of the three manufacturers, were analyzed in order to model metal release from a typical fixed orthodontic appliance. Metal release from the mesh of the brackets was prevented by bonding the brackets to 10 cm borosilicate glass rods with a conventional orthodontic adhesive (Transbond Supreme LV, 3M Unitek, Germany). Bands, on the other hand, though also typically cemented to the patient’s teeth, were investigated without bonding to a substrate since the area covered by adhesive varies from patient to patient. The grouped samples were, in each case, immersed in 50 ml of an artificial saliva solution and stored at room temperature in the dark. Exogenous metal contamination was avoided by using acid-cleaned PP tubes and sample handling in a class 10,000 clean room. In short, 0.5 ml samples were withdrawn after shaking at weekly intervals for 9 weeks and diluted to 5 ml with 0.6% v/v HNO_3_; pH was kept constant. Samples were collected up to half a year.

**Tab. 1:** Tested products and corresponding bulk chemical compositions **Tab. 1** Untersuchte Produkte und entsprechende chemische Zusammensetzung

Type	Vendor	Product Name	Alloy	Chemical composition, % by mass
BA	3M Unitek	Victory series, narrow contoured, 902	Stainless steel	18–20% Cr, 8–12% Ni, 2.0% Mn (max), 1.0% Si (max), balance: Fe
BA	Dentaurum	Dentaform	DIN 1.4303 (Cr, Ni austenitic stainless steel)	17–19% Cr, 11–13% Ni, 2.0% Mn (max), 1.0% Si (max), balance: Fe
BA	Dentaurum	Dentaform Snap	DIN 1.4541 (Cr Ni austenitic stainless steel stabilized with Ti)	17–19% Cr, 9–12% Ni, 2.0% Mn (max), 1.0% Si (max), 0.7% Ti (max), balance: Fe
BA	Ormco	Company Standard	AISI 305 (Cr, Ni austenitic stainless steel; similar to DIN 1.4303)	17–19% Cr, 10.5–13.0% Ni, 2.0% Mn, 1.0% Si, balance: Fe
BR	3 M Unitek	Victory Series, twin MBT	Stainless steel	18–20% Cr, 8–12% Ni, 2.0% Mn (max), 1.0% Si (max), balance: Fe
BR	Dentaurum	Discovery series, Roth 18	DIN 1.4303 (Cr, Ni austenitic stainless steel)	17–19% Cr, 11–13% Ni, 2.0% Mn (max), 1.0% Si (max), balance: Fe
BR	Ormco	Full-Size Diamond Twin series	AISI 303SE (austenitic stainless steel, comparable to DIN 1.4305)	17–19% Cr, 8–10% Ni, 2.0% Mn (max), 1.0% Si (max), 0.6% Mo (max), 0.5% Cu (max), 0.2% Co (max), 0.15–0.35% Se, balance: Fe
W	3M Unitek	Unitek Nitinol SuperElastic	Nitinol SuperElastic	66.4% Ni, 33.6% Ti
W	3M Unitek	Unitek Nitinol Heat-Activated	Nitinol Heat-Activated	66.4% Ni, 33.6% Ti
W	3M Unitek	Permachrome Standard	Permachrome (stainless steel)	16–18% Cr, 6–8% Ni, 2.0% Mn, 1.0% Si, balance: Fe
W	Dentaurum	Remanium	DIN 1.4310 (Cr, Ni austenitic stainless steel)	16–19% Cr, 6.0–9.5% Ni, 2.0% Mn (max), 2.0% Si (max), 0.8% Mo (max), balance: Fe
W	Dentaurum	Rematitan sl	Rematitan	50–60% Ni, 0.5% Fe (max), 0.1% Al (max), balance: Ti
W	Ormco	Ni–Ti	Ni–Ti	54,9% Ni, 0.20% Cr, balance: Ti
W	Ormco	Broad Arch	AISI 302 (Cr, Ni austenitic stainless steel)	17–19% Cr, 8–10% Ni, 2.0% Mn, 1.0% Si, balance: Fe

*BA* Bands, *BR* Brackets, *W* Wire

### Instrumentation

Metal ion release was quantitated using an inductively coupled plasma quadrupole mass spectrometer (ICP-MS, Elan DRC+, Perkin Elmer, USA). Polyatomic interferences from ^12^C^40^Ar on ^52^Cr were removed by bandpass tuning and using NH_3_ (BASF, Germany) as the reaction gas in the reaction cell. The ICP-MS was optimized for the highest signal-to-background ratio on ^52^Cr as reported elsewhere [29, 30].

^52^Cr, ^55^Mn, ^59^Co, ^60^Ni, and ^89^Y isotopes were recorded. Limits of quantitation (LOQ) calculated from the calibration function were 0.4 µg l^−1^ Cr, 0.4 µg l^−1^ Mn, 0.3 µg l^−1^ Co, and 0.5 µg l^−1^ Ni. The LOQs reported herein are already corrected for the ten-fold dilution each sample underwent prior to analysis.

Surface images were acquired using a scanning electron microscope (SEM; TESCAN VEGA3, Czech Republic) equipped with an energy dispersive X-ray microanalyzer (EDX).

### Statistics

IBM SPSS Statistics Version 22″ (2013) was used for descriptive and explorative statistical analysis of data. Descriptive and graphical data are expressed in mean values plus standard deviations. Differences were considered significant at *p* < 0.05.

## Results

Metal release from a total of 22 bands, 5 brackets, and 17 wires from three manufacturers (Table 1) was studied for a minimum of 44 days and, in some cases, nearly 2 months.

**Fig. 1:**
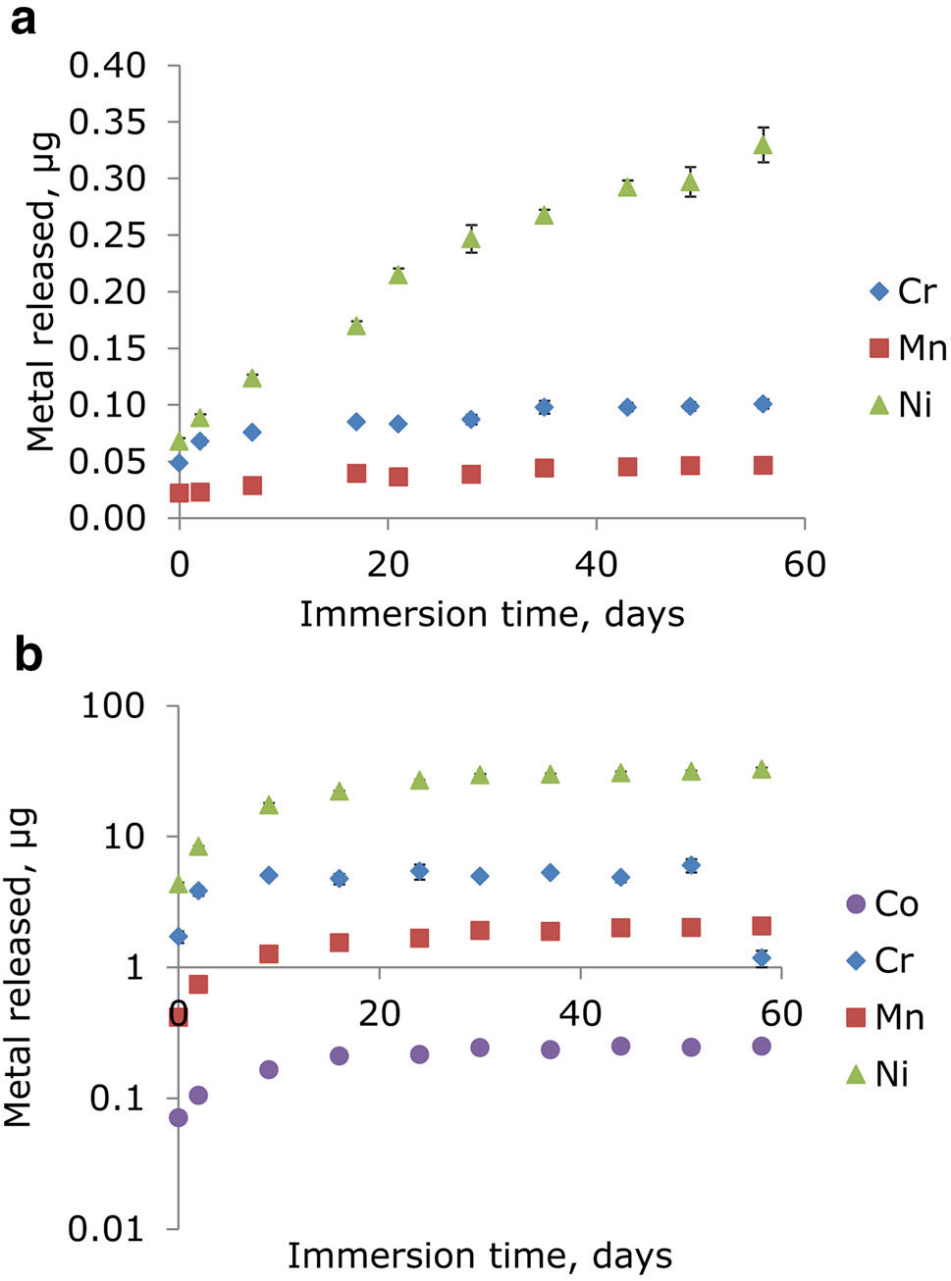
**a** Total Cr, Mn, Ni, and Co release from orthodontic brackets as a function of time during continuous immersion in 50 ml of an artificial saliva solution. Metal release from 20 brackets (3M Unitek, Victory Series, twin MBT, 017-452). Co was below the limit of quantification. **b** Total Cr, Mn, Ni, and Co release from orthodontic bands as a function of time during continuous immersion in 50 ml of an artificial saliva solution. Metal release from 4 bands (Ormco). Note use of logarithmic scale **Abb. 1 a** Gesamte Cr-, Mn-, Ni- und Co-Freisetzung aus kieferorthopädischen Brackets als Funktion der Zeit während der kontinuierlichen Lagerung in 50 ml künstlicher Speichellösung. Metallfreigabe aus 20 Brackets (3 M Unitek, Victory Series, Twin MBT, 017-452). Co- lag unterhalb der quantitativen Nachweisgrenze **b** Gesamte Cr-, Mn-, Ni- und Co-Freisetzung aus kieferorthopädischen Brackets als Funktion der Zeit während der kontinuierlichen Lagerung in 50 ml künstlicher Speichellösung. Metallfreigabe aus 4 Bändern (Ormco). Man beachte den logarithmischen Maßstab

The graphical representation of the release of Co, Cr, Mn, and Ni from selected brackets (Ormco full size diamond twin series) and bands (3 m Unitek Victory series, twin MBT, 017-452) over a period of nearly 2 months shown in Fig. 1 reveals three principle findings: First, the bands released markedly more Cr, Mn, and Ni than the brackets (a factor of 60, 44, and 98 more for Cr, Mn, and Ni, respectively). This implies that that bands dominate total metal ion release by a typical fixed orthodontic appliance (Co release from brackets was below the LOQ). A total of 32 ± 1 µg of nickel was for example released from the four bands during the study period. The second finding deducible from Fig. 1 is that the profile of Ni release by the bands was strikingly different from that of the brackets. Whereas the rate of nickel release from the brackets was constant during the 58 days of investigation, the bulk of the Ni release from the bands occurred during the first 9 days, afterwards leveling off and reaching a plateau. Third, Fig. 1 shows that the profiles of Mn and Cr ion release by brackets differed from the Ni release profile, plateauing after 35 days, and are thus more similar to the Ni ion release profile from bands.

The quantities of Co, Cr, Mn, and Ni released from all of the studied products (in each case during a 44 day period) are summarized in Tables 2 and 3. Metal amounts released from the 22 different analyzed band products (Table 2) varied greatly [18-fold (Co), 381-fold (Cr), 15-fold (Mn), and 50-fold (Ni)]. Moreover, huge variations were observed for bands made from the same alloy. The *t* test for independent samples showed significant differences between the groups. Due to different manufacturing processes and postprocessing curing, comparison of the materials between the companies is difficult.

**Tab. 2:** Co, Cr, Mn, and Ni release from orthodontic bands immersed in an artificial saliva solution for 44 days (total release and release profile). Metal release from 4 bands (22 different products). Total metal release is expressed as  the mean value ± standard uncertainty. Metal release profiles were grouped into three categories: “constant release” (α), “intermediate” (β), “saturation” (γ). Note: different custom attachments were welded onto the bands **Tab. 2** Co-, Cr-, Mn- und Ni-Freisetzung aus kieferorthopädischen Bändern, die 44 Tage in künstlicher Speichellösung gelagert waren (Gesamtfreisetzung und Verlaufsprofil). Metallfreisetzung aus 4 Bändern (22 unterschiedliche Produkte). Die Freisetzung von Metallen insgesamt ist angegeben als Durchschnittswert ± Standardabweichung, die Freisetzungsprofile wurden in 3 Kategorien eingeteilt: “konstante Freisetzung” (α), “intermediär” (β), “Sättigung” (γ). Man beachte, dass auf den Bändern unterschiedliche Attachments aufgebracht waren

Vendor	Product name	Co, µg	Cr, µg	Mn, µg	Ni, µg
3M Unitek	Victory Series, Narrow Contoured, 902	0.030 ± 0.001 (γ)	0.70 ± 0.05 (γ)	0.256 ± 0.008 (γ)	5.9 ± 0.2 (γ)
	Custom attachments 1				
3M Unitek	Victory Series, Narrow Contoured, 902	0.178 ± 0.005 (γ)	6.4 ± 0.4 (γ)	0.39 ± 0.01 (γ)	14.9 ± 0.5 (γ)
	Custom attachments 2				
3M Unitek	Victory Series, Narrow Contoured, 902	0.229 ± 0.007 (β)	1.33 ± 0.09 (β)	1.77 ± 0.05 (β)	22.0 ± 0.7 (β)
	Custom attachments 3				
3M Unitek	Victory Series, Narrow Contoured, 902	0.035 ± 0.002 (β)	0.084 ± 0.004 (β)	0.139 ± 0.004 (β)	0.67 ± 0.02 (β)
	Custom attachments 4				
3M Unitek	Victory Series, Narrow Contoured, 902	0.036 ± 0.002 (β)	0.037 ± 0.002 (β)	1.65 ± 0.05 (β)	1.95 ± 0.06 (β)
	Custom attachments 5				
3M Unitek	Victory Series, Narrow Contoured, 902	0.036 ± 0.002 (β)	0.15 ± 0.01 (β)	0.228 ± 0.007 (β)	2.04 ± 0.06 (β)
	Custom attachments 6				
Dentaurum	Dentaform, custom attachments 7	0.054 ± 0.002 (α)	1.72 ± 0.08 (γ)	0.81 ± 0.02 (α)	3.5 ± 0.1 (α)
Dentaurum	Dentaform, custom attachments 8	0.55 ± 0.02 (α)	14.1 ± 0.6 (γ)	3.4 ± 0.1 (α)	25.2 ± 0.8 (α)
Dentaurum	Typ 8780 Gr 22 & Gr23	0.106 ± 0.003 (β)	0.29 ± 0.01 (β)	0.63 ± 0.02 (β)	5.4 ± 0.2 (β)
Dentaurum	Typ 8802 Gr 22 & Gr23	0.048 ± 0.001 (β)	0.084 ± 0.006 (β)	0.31 ± 0.01 (β)	2.54 ± 0.08 (β)
Dentaurum	Typ 8790 Gr 22 & Gr23	0.150 ± 0.005 (β)	2.8 ± 0.2 (β)	0.99 ± 0.03 (β)	8.1 ± 0.2 (β)
Dentaurum	Typ 8812 Gr23	0.164 ± 0.005 (β)	0.56 ± 0.04 (β)	1.86 ± 0.06 (β)	9.8 ± 0.3 (β)
Dentaurum	882-004-022	0.042 ± 0.001 (β)	0.051 ± 0.002 (β)	0.58 ± 0.02 (β)	1.78 ± 0.05 (β)
Dentaurum	883-005-032	0.136 ± 0.004 (β)	0.21 ± 0.01 (β)	0.78 ± 0.02 (β)	4.9 ± 0.2 (β)
Dentaurum	884-004-032	0.045 ± 0.002 (β)	0.086 ± 0.006 (β)	0.72 ± 0.02 (β)	1.52 ± 0.05 (β)
Dentaurum	885-004-032	0.207 ± 0.006 (β)	0.32 ± 0.02 (β)	0.76 ± 0.02 (β)	3.8 ± 0.1 (β)
Ormco	Custom attachments 9	0.249 ± 0.008 (γ)	5.3 ± 0.2 (α)	2.01 ± 0.06 (α)	30.5 ± 0.9 (α)
Ormco	Custom attachments 10	0.102 ± 0.003 (γ)	0.47 ± 0.03 (γ)	2.07 ± 0.06 (α)	4.4 ± 0.1 (γ)
Ormco	001-1000	0.080 ± 0.002 (β)	0.21 ± 0.01 (β)	1.15 ± 0.03 (β)	4.8 ± 0.1 (β)
Ormco	001-1001	0.054 ± 0.002 (β)	0.165 ± 0.006 (β)	1.61 ± 0.05 (β)	0.62 ± 0.02 (β)
Ormco	001-1006	0.164 ± 0.005 (β)	0.41 ± 0.01 (β)	0.99 ± 0.03 (β)	4.3 ± 0.1 (β)
Ormco	001-1007	0.43 ± 0.01 (β)	0.083 ± 0.006 (β)	0.76 ± 0.02 (β)	2.58 ± 0.08 (β)

Metal release from 4 bands (22 different products). Total metal release is expressed as the mean value ± standard deviation. Metal release profiles were grouped into three categories: “constant release” (α), “intermediate” (β), “saturation” (γ). Note: different custom attachments were welded onto the bands

**Tab. 3:** Metal release from wires (17 different products) and metal release from orthodontic brackets (5 different products) **Tab. 3** Metallfreisetzung aus Drähten (17 verschiedene Produkte) sowie aus kieferorthopädischen Brackets (5 unterschiedliche Produkte)

Vendor	Alloy	Product Name	Co, µg	Cr, µg	Mn, µg	Ni, µg
3M Unitek	Nitinol Heat-Activated	4297-913	<LOQ	<LOQ	<LOQ	0.40 ± 0.02 (γ)
3M Unitek	Nitinol Heat-Activated	4296-991 4296-992	<LOQ	<LOQ	<LOQ	0.208 ± 0.006 (γ)
3M Unitek	Nitinol Heat-Activated	9296-611 9296-612	<LOQ	<LOQ	<LOQ	0.094 ± 0.003 (α)
3M Unitek	Nitinol SuperElastic	4296-912 4296-911	<LOQ	<LOQ	<LOQ	0.17 ± 0.01 (α)
3M Unitek	Nitinol SuperElastic	4297-833 4297-834	<LOQ	<LOQ	<LOQ	0.27 ± 0.01 (γ)
3M Unitek	Nitinol SuperElastic dimpled	9296-914 9296-913	<LOQ	<LOQ	<LOQ	0.15 ± 0.01 (α)
3M Unitek	Permachrome	300-018	<LOQ	0.045 ± 0.001 (γ)	0.037 ± 0.001 (γ)	0.049 ± 0.002 (γ)
3M Unitek	Permachrome	299-185	<LOQ	<LOQ	0.026 ± 0.001 (γ)	0.041 ± 0.001 (γ)
Dentaurum	DIN 1.4310	765-201-00 765-301-00	<LOQ	0.033 ± 0.001 (γ)	0.059 ± 0.002 (γ)	0.107 ± 0.003 (γ)
Dentaurum	DIN 1.4310	767-101-00 767-201-00	<LOQ	0.027 ± 0.001 (γ)	0.064 ± 0.002 (α)	0.095 ± 0.003 (γ)
Dentaurum	Rematitan	766-080-00 766-081-00	<LOQ	<LOQ	<LOQ	2.47 ± 0.07 (γ)
Dentaurum	Rematitan	766-082-00 766-083-00	<LOQ	<LOQ	<LOQ	1.32 ± 0.04 (γ)
Ormco	AISI 302	210-0026	<LOQ	<LOQ	0.028 ± 0.001 (α)	0.057 ± 0.004 (γ)
Ormco	AISI 302	210-0701	<LOQ	<LOQ	0.113 ± 0.003 (α)	0.076 ± 0.003 (γ)
Ormco	Ni–Ti	205-0009	<LOQ	<LOQ	<LOQ	0.269 ± 0.008 (α)
Ormco	Ni–Ti	205-0001	<LOQ	<LOQ	<LOQ	0.098 ± 0.007 (γ)
Ormco	Ni–Ti	210-0502	<LOQ	<LOQ	<LOQ	0.152 ± 0.009 (γ)
3 M Unitek	Stainless steel	Victory Series, twin MBT, 017-452	<LOQ	0.098 ± 0.003 (γ)	0.045 ± 0.001 (γ)	0.292 ± 0.009 (α)
3M Unitek	Stainless steel	Victory Series, twin MBT, 017-552	<LOQ	0.095 ± 0.003 (α)	0.054 ± 0.002 (γ)	0.281 ± 0.008 (α)
Dentaurum	DIN 1.4303	Discovery series, Roth 18, Part Numbers 790-141-00 & 790-141-00 & 790-104-00 & 790-102-00 & 790-163-00 & 790-164-00 & 790-103-00 & 790-105-00 & 790-142-00 & 790-142-00	<LOQ	0.035 ± 0.002 (γ)	<LOQ	0.119 ± 0.006 (γ)
Dentaurum	DIN 1.4303	Discovery series, Roth 18, Part Numbers 790-145-00 & 790-143-00 & 790-108-00 & 790-107-00 & 790-107-00 & 790-107-00 & 790-107-00 & 790-109-00 & 790-144-00 & 790-146-00	<LOQ	0.034 ± 0.001 (γ)	<LOQ	0.149 ± 0.005 (γ)
Ormco	AISI 303SE	Full-Size diamond twin series, Part Numbers 347-2001 & 340-1404 & 340-1500 & 347-1208 & 347-1308 & 340-1504 & 340-1505 & 347-2101 & 347-2018 & 347-2118	0.026 ± 0.001 (γ)	0.132 ± 0.004 (γ)	0.274 ± 0.008 (α)	1.61 ± 0.05 (γ)

**Fig. 2:**
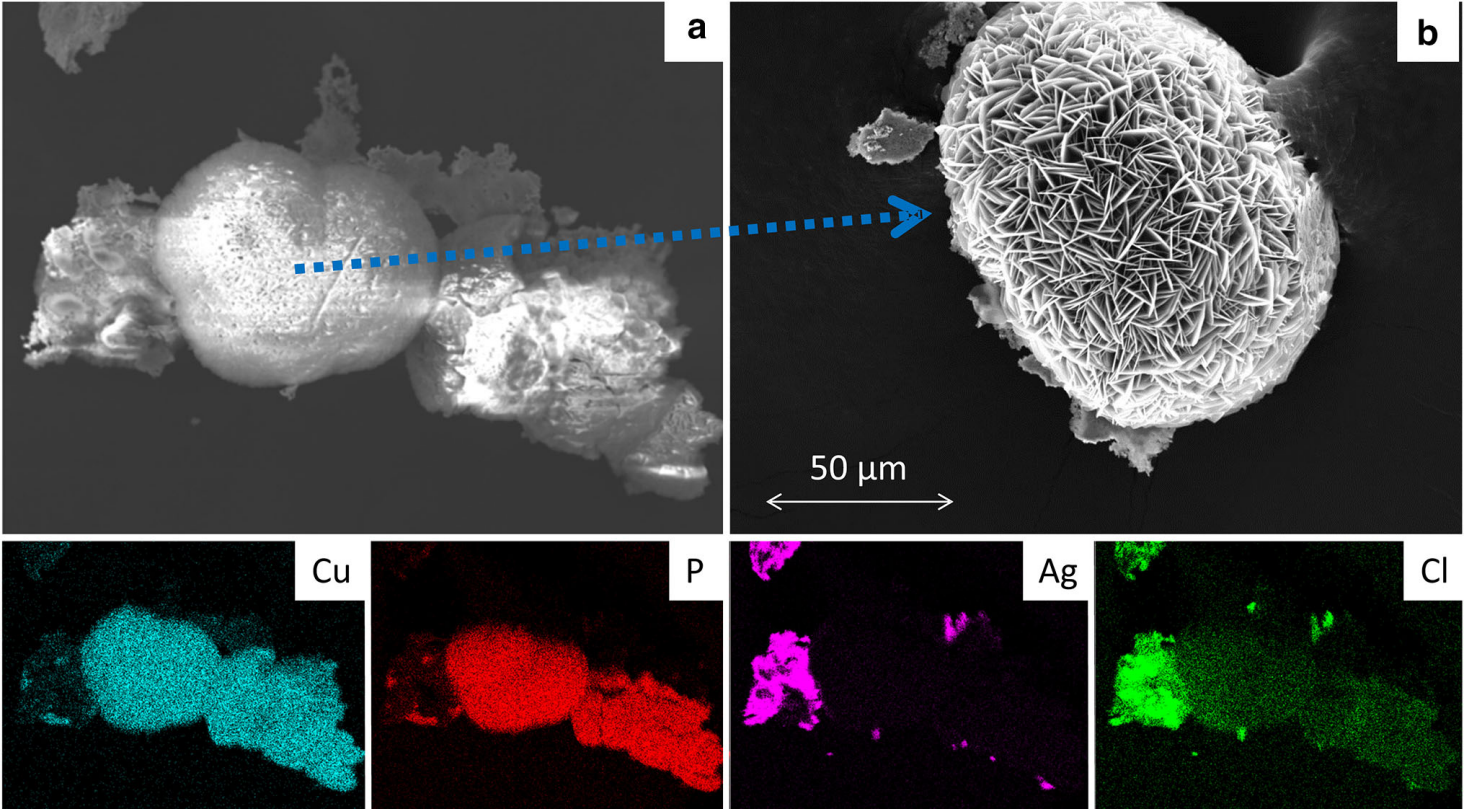
REM images and EDX maps of turquois deposits formed on bands after 6-month immersion in artificial saliva solution. **a** Typical REM image of a deposit together with EDX elemental maps of Cu, P, Ag, and Cl. No nickel could be detected by EDX. **b** Enlarged REM image of a structure similar to the center of image in **a**. **b** Image recorded after gold-sputtering for better image quality. Note that images **a** and **b** are two different particles **Abb. 2** REM-Bilder und EDX-Abbildungen von türkisfarbenen Ablagerungen auf den Bändern nach 6-monatiger Lagerung in künstlicher Speichellösung. **a** Typisches REM-Bild einer Ablagerung zusammen mit EDX-Elementarhochbildkarten von Cu, P, Ag und Cl. Nickel konnte von EDX nicht erkannt werden. **b** Vergrößertes REM-Bild einer Struktur ähnlich der Bildmitte in **a**. Die Abbildung **b** wurde zwecks beserer Bildqualität nach Goldpulverisierung aufgezeichnet. Man beachte, dass es sich in den Abbildungen **a** und **b** um 2 verschiedene Partikel handelt

*<LOQ* below quantitation limit, *α* constant release denoting a profile like the one observed for Ni in Fig. 1, *γ* saturation to describe plateau-like profiles like the ones shown in Fig. 2, *β* intermediate release designating a release profile that showed signs of saturation but which did not reach saturation during the study period

It is also interesting to note that Co was released by all of the tested bands although this metal was not listed as an alloy constituent by the vendor (Table 1). The quantities of Co, Cr, Mn, and Ni released from the five different brackets that were analyzed (Table 3) were, in general, about one to two orders of magnitude lower than from the bands. A detectable, yet low level of Co was released by only one product (Ormco) which was traced back to the Co-containing alloy. Among the 17 tested wire products (Table 3), the amount of Ni released was generally comparable with that released by brackets, while the quantities of Co, Cr, and Mn were significantly lower.

Tables 2 and 3 also list the profiles of Co, Cr, Mn, and Ni release for each of the tested products (except when the amount of a given ion released was below the limit of quantitation). To facilitate comparison, three broad types of release profile were defined using the findings shown in Fig. 1 as a basis, namely, “constant release (α)” denoting a profile like the one observed for Ni in Fig. 1, “saturation (γ)” to describe plateau-like profiles like the ones shown in Fig. 2, and “intermediate (β)” release designating a release profile that showed signs of saturation but which did not reach saturation during the study period.

Most bands (Table 2) exhibited a β-type release profile (16/22 products) for Co, Cr, Mn, and Ni. Of the other six products, two exhibited a γ-type profile for all metals, while the remaining four had a “mixed profile” (i.e., an α-type profile for one or more metals as well as a γ-type profile for at least one metal). In marked contrast, brackets and wires exhibited either α-type or γ-type metal release (including uniform and “mixed release”).

The immersion in artificial saliva solution of some bands was continued for 6 months. At the end of this period some bands showed signs of corrosion and tiny turquoise spots became visible. In cases in which the attachments were spot welded to the band, the initially bright metal surface around the welding spots became matt.

REM images of the brittle turquoise spots revealed a heterogeneous mixture of crystals. As shown in Fig. 2a, two distinct structures dominated: a well-ordered arrangement of flat sheets (see Fig. 2b for an enlarged image) containing high concentrations of copper and phosphorous and a seemingly chaotic arrangement of very small crystals with indistinct structures. These regions were rich in silver and chlorine. It is important to note that no nickel was detected by EDX. Iron on the other hand was present in regions of high chlorine but of low silver concentration, thus, filling the gaps in the silver map.

The high copper and phosphorous content of the flat, sheet-like crystals indicates the presence of copper (II) phosphate. Indeed, Cu_3_ (PO_4_)_2_·2H_2_O is a greenish salt of low solubility (0.005 g l^−1^ in water at 293 K) [25] that forms the observed crystal structure, as reported by Wu and Shi [32]. In regions of high silver and chlorine concentration the formation of silver chloride seems reasonable. AgCl is also poorly soluble in water (0.002 g l^−1^ at 298 K) [15].

The deposition of copper (II) phosphate was observed on all bands with attachments from Dentaurum and Ormco, though no turquoise deposits were encountered on 3 M Unitek products. Ormco states that some attachments (buccal tubes) are made from a martensitic precipitation hardening, chromium, nickel, copper stainless steel (DIN 1.4542) containing 15.5–17.5% Cr, 3–5% Cu and Ni, 1% Mn and Si, 0.15–0.45% Nb in an iron matrix, whereas Dentaurum uses cobalt alloys (ISO 6871-1 [1994]) for this purpose in some products.

These results indicate that the release of metals is not dominated by the composition of the band bulk metal matrix but rather by the attachments, their composition, and mode of attachment to the band.

## Discussion

It is well known that the release of metals from metallic substrates is not a straightforward process. López-Alías et al. [19] for instance reported that, while alloy composition seems to play an important role in metal release, the differences in the amounts of nickel released by three alloys containing a high percentage of nickel were nonetheless surprising.

The nonlinear release of Co, Cr, Mn, and Ni from many commercial brackets and bands over a period of nearly two months observed in the present study raises questions concerning the use of previously published constant release rates in units of ng cm^−2^ day^−1^ to estimate metal exposure associated with orthodontic treatment. Of the 64 release profiles recorded, 43 showed a plateau-like temporal profile. The use of constant release rates will, thus, clearly underestimate metal intake during the first couple of days and overestimate exposure during the remainder of the treatment which is usually several months long.

The variable metal release profiles observed are probably largely due to differing degrees of surface passivation of the tested components since formation of a tight passivation layer would impede further metal release. The Ni release profile for brackets in Fig. 1 is not a linear function and can be interpreted as showing the onset of a saturation as exhibited by Ni release from bands during the first 9 days.

Another interesting finding was the release of Co by bands, despite the fact that Co was not listed as an alloy constituent. Unlike brackets and wires, bands with attachments are commonly custom-made rather than standardized products and the alloy used to manufacture the band does not necessarily need to be the same as the alloy used to make the attachment. The large differences in the quantities of Co, Cr, Mn, and Ni released by bands of the same type (Table 2) can, therefore, be potentially attributed to the different attachments welded onto the bare bands.

The use of a constant pH value in the present study avoided additional influences of variable pH on metal release. Kuhta et al. [18] reported that a reduction of salivary pH from 6.75 to 3.5 can increase the release of metal ions from orthodontic appliances by up to 100-fold. Low pH values moreover reduced the resistance of dental alloys to corrosion [12].

Barret et al. [4] showed that NiTi arch wires released more Ni than their stainless steel counterparts. This result was replicated by the present study, though, with the exception of the rematitan product to a lesser degree. The tested arch wires released quantities of Ni comparable to the studied brackets though significantly lower amounts of Co, Cr, and Mn. Jia et al. [14] concluded that the maximum amount of nickel released from all tested arch wires was 700 times lower than the concentrations necessary to elicit cytotoxicity reactions.

Arndt et al. [3] showed that the maximum release of Ni ions from NiTi orthodontic wires was two orders of magnitude below the daily dietary intake level. Mechanical and thermal loading increase Ni release by a factor of 10–30, the highest being 8.0 µg/day, the lowest 0.5 µg/day.

Amini et al. [1] reported that the mean salivary nickel (Ni) content in subjects with and without a fixed orthodontic appliance was 18.5 ± 13.1 versus 11.9 ± 11.4 ng/ml and for chromium 2.6 ± 1.6 ng/ml in the study group and 2.2 ± 1.6 ng/ml in the control group. They concluded that the presence of fixed orthodontic appliances leads to an increased concentration of metal ions in salivary secretions.

Petoumenou et al. [23] found a slight but significant increase in the salivary nickel concentration of 78 µg l^−1^ immediately after placement of the bands, brackets, and the NiTi archwires, compared with the pretreatment value of 34 µg l^−1^. This effect decreased within 10 weeks or even earlier (2–8 weeks). Other authors [7] described a release of Ni and Cr of about 40 µg Ni and 36 µg Cr per day when a full-mouth orthodontic appliance was used, and even in case of recycled orthodontic brackets, the total ion release did not exceed the recommended daily intake. This is noteworthy because most orthodontic brackets are made of austenitic stainless steel and any heating leads to weakening of the structure through partial disintegration [12]. In this investigation, a total of up to 43 ± 1 µg Co, Cr, Mn, and Ni were released from the four bands after 44 days and this magnitude was far higher compared to the bracket value. In this setting, the whole surface of the band was in contact with the artificial saliva and as a consequence a possible overestimation of ion release is possible. On the other hand, the highest corrosion was seen on the welded attachments, which seem to be the main source for ion release. The release of metals from the tested arch wires was comparable with the brackets for nickel, but the release of cobalt, chromium, and manganese was significantly lower.

Of course, there are limitations of in vitro and in vivo studies, as not all in vivo influences can be simulated by in vitro experiments and on the other side in vivo investigations are limited by additional effects, such as saliva composition, saliva fluid rate, thermal exposition, pH differences, and protecting impacts. For instance, recent studies focusing on the alterations of NiTi wires found that the material surfaces were coated by intraorally formed proteinaceous integuments that mask the alloy surface topography to an extent dependent on an individual patient’s oral environment and exposure period. This biofilm causes precipitate on the wire surface and the mineralized regions may provide a protective effect especially under low pH conditions with increased risk of corrosion [7].

Normally the pH value of the saliva ranges from 6.5–6.9 at rest, up to 7.0–7.2 after stimulation. Stimulation can change the protein composition of saliva and nickel associates with proteins, affecting the nickel concentration. In addition, the autonomic nervous system emotional state influences the saliva flow rate [23], resulting in further uncertainties in the saliva metal ion concentration determination.

Ortiz et al. [22] proved that titanium brackets and tubes were the most biocompatible when comparing three different alloys. They found that the greatest damage to cellular DNA was caused by stainless steel alloy, followed by nickel-free alloy. Contrary to this, titanium alloy induced increased cellular viability and did not damage cellular DNA.

For patients, who are not Ni hypersensitive at the start of orthodontic treatment, the risk for orthodontic-derived Ni allergy is extremely low because of the slow long-term release from orthodontic appliances [7, 31].

On the other hand, Kerosuo et al. [17] concluded that the nickel and chromium concentrations of saliva are not significantly affected by fixed orthodontic appliance during the first month of treatment. But local concentrations of nickel on the oral mucosa might be sufficient to elicit allergic reaction [10]. In such a case the cobalt chromium bracket, which is essentially nickel free, should be used [12].

Taken together, the present metal release data provide evidence that metal release from a typical orthodontic appliance is small compared to the potential uptake from dietary sources. Even in the worst case scenario, i.e., with an appliance comprising those bands, brackets, and wires found to release the highest quantities of the four metals, total additional metal exposure after 44 days would be only 0.58 µg Co, 14 µg Cr, 3.8 µg Mn, and 34.6 µg Ni. These values seem to be small when comparing the potential uptake of these metals with the uptake from dietary sources.

The release of Ni from fixed orthodontic appliances has been reported to be related to both the composition and the method of manufacture of the appliance components and not to Ni content [11]. This is confirmed by the present study which found that the magnitude of metal release and release profile varied among brackets, wires, and bands. The amount of metal released and the release profile seem to be component specific rather than to be specific for properties of the individual metallurgical alloy.

## Conclusion

From this study, it appears that bands are the main source of the Co, Cr, Mn, and Ni released by a typical orthodontic appliance, followed by brackets and wires. Moreover, these metals are not released as a linear function of time. Whereas some orthodontic components continue to release metals, other seem to become passivated after a certain time, stopping further metal release. Consequently, a normalization to µg cm^−3^ day^−1^ is not useful. Variable metal release by bands appears to depend strongly on the nature of the attachments welded to the band and the weld itself since products comprising the same band and different attachments liberated variable metal quantities. This hypothesis is supported by the fact that cobalt proved to be an alloy constituent of the band attachments but not of the bands per se. While our data are consistent with heavy metal release by orthodontic materials at levels well below typical dietary intake, we nevertheless recommend the use of titanium brackets and replacement of the band with a tube in cases of severe Ni or Cr allergy.
